# Potential of Hairless Canary Seed as a Food-Based Remedy for Celiac Disease and Diabetes †

**DOI:** 10.3390/foods14173011

**Published:** 2025-08-28

**Authors:** El-Sayed M. Abdel-Aal, Tamer H. Gamel

**Affiliations:** Agriculture and Agri-Food Canada, Guelph Research and Development Centre, Guelph, ON N1G 5C9, Canada; tamer.gamel@agr.gc.ca

**Keywords:** glabrous canary seed, gluten-free foods, bioactive peptides, phenolic compounds, carotenoids, chronic diseases

## Abstract

Hairless canary seed (*Phalaris canariensis* L.) can play significant roles in human health and nutrition due to its unique nutrient profile. It belongs to the *Gramineae* family similar to common cereal grains like wheat, rice and corn. On the other hand, the traditional canary seed is characterized by the presence of silicified spicules or hairs on the hulls of the kernel that could pose health hazards to humans. The hairless canary seed was developed in Canada by a conventional breeding program to mitigate the health concerns associated with the silicified hairs. The hairless grain is silica free, i.e., totally glabrous, and is granted regulatory food approvals by Health Canada and US-FDA. The hairless grain holds a great potential as a whole grain functional food ingredient due to its unique nutritional and functional attributes. As a cereal grain, it is rich in protein that is non-gluten and exceptionally high in tryptophan and bioactive peptides. The grain also contains reasonable amounts of carotenoids, polyphenols, and healthy unsaturated oil. Because of these special characteristics, it is considered a promising nutritious and therapeutic food. This review provides insights into the potential of hairless canary seed as a functional ingredient in products designed to mitigate oxidative stress, diabetes and celiac disease and/or to improve vision and cognition.

## 1. Introduction

**Figure 1:**
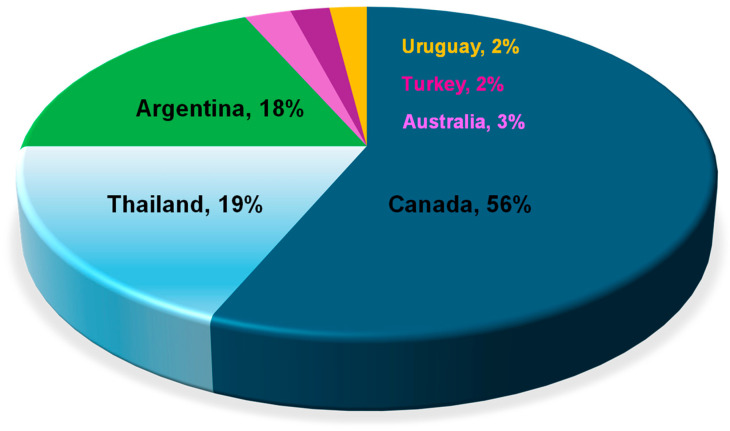
Major country producers of canary seed in the world, the total world production, 199,000–304,000 tons. Source: FAO 2023 [1].

Food shortages are a major global issue particularly in regions largely impacted by climate change, poverty and/or political conflict. A number of mitigation strategies have been underway to combat this challenge in order to secure more food supplies for the growing world population. Non-traditional crops that are high in the essential nutrients especially protein and energy could play significant roles in those strategies as sustainable food sources. Among them, canary seed (*Phalaris canariensis* L.) stands out for its short growing cycle, adaptability to marginal soils, and potential to diversify crop rotations, making it a promising complementary cereal to support global food security. In addition, it is relatively high in protein and calories and can provide a new source of nutritious food. According to the FAO data on crop production in 2023, Canada is the main global canary seed producer at about 56% of the world production, followed by Thailand (19%) and Argentina (18%) (Figure 1) [1]. Other main producing countries include Australia (3%), Turkey (2%) and Uruguay (2%). Canada is also the leading exporter of canary seed accounting for >80% of the world exports. In contrast, Mexico, Belgium, Brazil, Spain, USA and Colombia are the major importing countries. Over the past 10 years, the global canary seed production was between 199,000 and 304,000 tons, being the highest in 2020 and the lowest in 2023 [1]. Currently, the primary market for canary seed is birdseed which is a small market, thus only small amounts of canary seed are produced globally.

**Figure 2:**
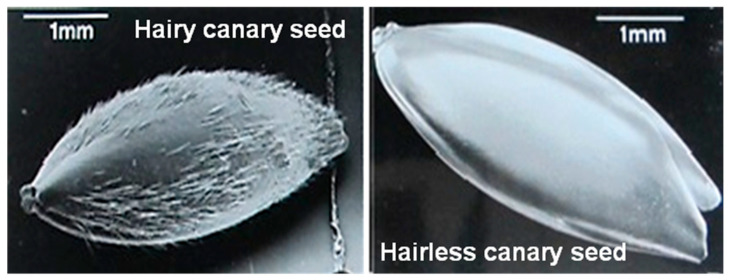
Scanning electron microscopy photomicrographs of hairy and hairless canary seed. Source: Abdel-Aal et al., 1997 [2].

The traditional canary seed is considered unsafe as a food for human because of the presence of tiny silicified hairs known as trichomes or spicules on the surface of the kernel hulls, i.e., hairy or pubescent canary seed (Figure 2) [2]. The hairs are severe skin irritants and potentially carcinogenic being linked to esophagus cancer [3]. During the production of canary seed, the hairs could contaminate other grains grown close to the canary seed field. The spicules may also pollute canary seed groats during dehulling of canary seed which pose potential health threats to consumers. The hairless canary seed was developed in Canada by a conventional breeding program at the University of Saskatchewan to avoid skin irritation of farmers by hairs during harvesting and other potential health issues [2]. The entire removal of the harmful silicious spicules (e.g., silica free, Figure 2) makes hairless canary seed a safe food and opens up new markets beyond the existing birdseed market.

Canary seed is a true cereal grain belonging to the same family (*Poaceae* or *Gramineae*) as other cereals such as wheat, rice, corn and oat. Both canary seed and oat exhibit comparable nutritional properties as they contain non-gluten proteins and relatively high amounts of oil compared to other cereals. In fact, they belong to the same tribe *Poeae*. The grain of hairless canary seed is covered or hulled (i.e., the hulls remain attached to the grain after harvesting process), and once dehulled for food use it is called groats [2]. The hairless grain holds a great potential as a whole grain functional food ingredient due to its unique nutritional and functional attributes. These include but not limited to its high contents of non-gluten protein, unsaturated oil, polyphenols and carotenoids. Since our previous report which has been published in 2021 focusing on the nutritional properties of hairless canary seed and its components [4], more information has been reported on the role of canary seed in human health. The current review is intended to provide the most recent knowledge and insights into the potential of hairless canary seed as a functional ingredient for products designed to mitigate chronic diseases such as oxidative stress, obesity, diabetes and celiac disease, and/or to improve vision and cognition.

## 2. Hairless Canary Seed as a Novel Food

**Figure 3:**
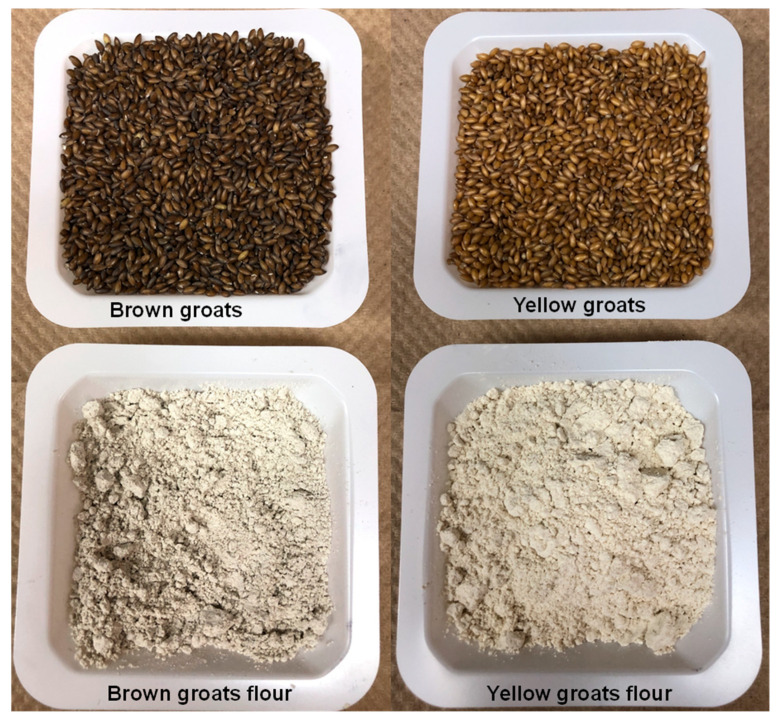
Appearance of yellow and brown hairless canary seed groats and their flours.

**Figure 4:**
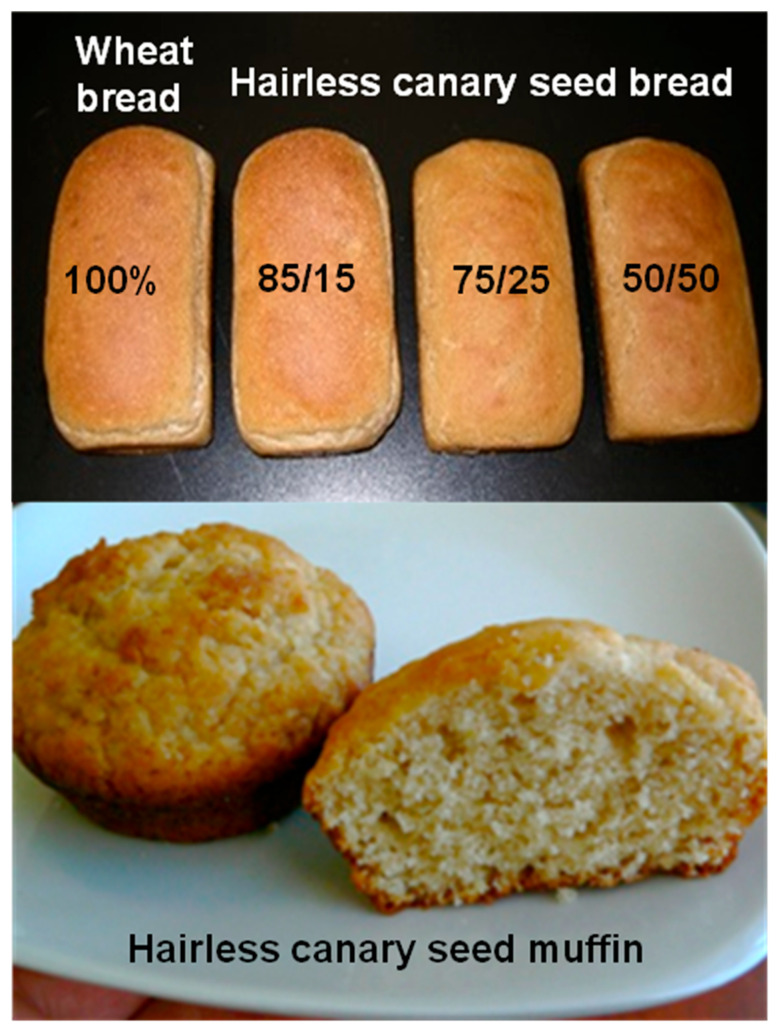
Appearance of hairless canary seed breads and muffins. Breads are made from 100% wheat flour (control) and blends of wheat and hairless canary seed flour at ratios of 85/15, 75/25 and 50/50 (*w*/*w*).

The US-Food and Drug Administration (FDA) and Health Canada conducted a thorough scientific assessment of human consumption of hairless canary seeds. In 2015, the hairless canary seed was granted GRAS status (Generally Reorganized As Safe, GRAS Notice No. GRN 000529) from the US-FDA [5], and in 2016 was accepted by Health Canada as a novel food [6]. The hairless or glabrous variety was developed to entirely eliminate the tiny silicious hairs that rendered the grain unsuitability for food use. The hairless variety is silica free and safe for human consumption. As a cereal grain, hairless canary seed has comparatively high contents of protein (22.7%) and oil (7.7%), with a comparable level of starch (57.2%) along with unique qualities for each component [4]. For instance, the protein is exceptionally high in tryptophan, averaging 2.8 g per 100 g protein [2,4] and bioactive peptides [7]. The oil belongs to the group “oils low in palmitic acid and high in oleic and linoleic acids” and contains high amounts of unsaturated fat, on average, linoleic (53.8%), oleic (24.2%) and linolenic acid (2.8%), and low amount of saturated fat, palmitic (11.5%) [8,9]. The starch granules in hairless canary seed also exhibit special properties being small and uniform, and form rigid gels with high stability under cold or frozen storage [10,11,12]. Hairless canary seed is also a good source of micronutrients, including iron, magnesium, and zinc, and contains antioxidant compounds such as phenolic acids and flavonoids [13,14,15]. Such nutrient profile and compositional properties make hairless canary seed a promising ingredient in food formulations to fill the food gap especially with the steadily increase in the global population. In fact, several review articles point out the role of hairless canary seed as a novel food ingredient in bakery, gluten-free and functional food applications [16,17]. It also holds a great potential as a whole grain functional food ingredient [4,13] and/or sustainable source of starch, protein and oil [18,19]. Figure 3 shows the appearance of yellow and brown hairless canary seed groats and their flours. Additionally, the hairless canary seed foods contain reasonable amounts of carotenoids [14] and phenolic acids and exhibit reasonable antioxidant activities [15]. Hairless canary seed has been processed into a variety of food products including bread, muffin, pasta, and others. Examples of hairless canary seed food prototypes that were made in house at the Guelph Research and Development Centre are given in Figure 4. In general, hairless canary seed is considered a nutritious cereal grain based on its compositional properties having a great potential in food applications.

## 3. Uniqueness of Hairless Canary Seed Proteins

As mentioned above canary seed proteins possess high amount of tryptophan showing their potential as blending ingredients in foods designed to boost the daily consumption of tryptophan. This essential amino acid provides a number of positive functions to boost the health of animals and humans [20,21]. For instance, it is involved in gut-brain functions, bone health, immune modulation and mitochondrial function [22]. It is also vital for the biosynthesis of a number of biologically-active compounds such as serotonin, melatonin, kynurenine and niacin [22]. In a study on an aqueous extract of canary seed, it has been found to act as a volume-independent antihypertensive mediator in normotensive and hypertensive rats due to the presence of kynurenine, a tryptophan metabolic derivative [23]. Kynurenine produces a range of biologically-active compounds that invoke important functions in human. The proteins in canary seed also have high concentrations of phenylalanine, arginine and cysteine along with low concentrations of lysine and proline which suggests unique functionality for canary seed proteins [18]. Th ratio of arginine-to-lysine or glycine-to-methionine have been found to induce beneficial effects on lipid profile and plasma levels of asymmetric dimethylarginine (ADMA) and symmetric dimethylarginine (SDMA) and homocysteine in hypercholesterolemic rats [24]. The low ratio of lysine-to-arginine in canary seed proteins would warrant investigations to determine its potential hypocholesterolemia if any. In general, the special characteristics of amino acid composition in hairless canary seed could make it a good choice in the development of functional foods.

Animal studies have shown that protein quality of hairless canary seed is equivalent to other common cereals [25,26,27]. The proteins in hairless canary seed exhibit ileal protein digestibility in broiler chicks similar to wheat, corn, sorghum and peas [25]. The study has also shown a high apparent ileal amino acid digestibility of canary seed protein with tryptophan exhibiting the highest digestibility (93%), while lysine exhibit lowest digestibility. Furthermore, canary seed has equal nutritional value in comparison with other grains fed to broiler chicks [26]. In a study on pigs, it has been reported that the protein digestibility increases with the addition of canary seed to the diets with a diet containing 25% canary seed exhibiting the highest growth rates in pigs along with a crude protein digestibility of 78% [27]. To the best of our knowledge, there are no human studies on the assessment of hairless canary seed proteins, but a few in vitro studies have shown comparable protein quality to other plant proteins [2,28]. A study reported in vitro protein digestibility of 84% for canary seed using a multi-enzymes method including trypsin, chymotrypsin and peptidase [2]. In a recent study, the in vitro protein digestibility of hairless canary seed measured by two methods, pH-drop and INFOGEST (international network of excellence on the fate of food in the gastrointestinal tract) is fairly lower (76.2–77.3%) than wheat (82.5%) and higher than oat (75.0%) [28]. The values obtained by the pH-drop method are higher than those measured by the INFOGEST, and the yellow-grained varieties exhibit better overall digestibility than the brown ones. The in vitro protein digestibility of roasted (176 °C for 12 min) and boiled (12 min) hairless canary seed flour determined by gastric, duodenal and sequential gastric-duodenal protocols has been found to be higher than that of the raw flour [29]. The proteins are more digestible under the sequential gastric-duodenal protocol compared with that of the gastric or duodenal digestion alone. Roasting of canary seed exhibits a larger impact on protein structure than boiling as indicated by protein subunits in the roasted samples resulting in greater in vitro protein digestibility than the boiling process [29]. The IVPD (in vitro protein digestibility) and IVPDCACS (in vitro protein digestibility corrected amino acid score) of yellow and brown hairless canary seed flours have a range of 78–81% and 30–31%, respectively [30]. However, the protein isolates exhibit higher IVPD (86–87%) and IVPDCACS (56–64%) values than flours. In general, lysine is the first limiting amino acid in hairless canary seed flours, while histidine or threonine is the first limiting amino acid in the protein isolate subject to the canary seed variety. The study indicates more research is still needed to characterize hairless canary seed flours and isolates in terms of protein quality and essential amino acid composition. It appears that hairless canary seed flours have comparable or superior in vitro protein digestibility to other cereal grains but human studies are essential to demonstrate the unique properties of hairless canary seed amino acid composition, and protein quality, digestibility and absorption.

## 4. Potential of Hairless Canary Seed in Celiac Disease Remedy

Cereal grains that their proteins form gluten such as wheat and durum or exhibit gluten sequence homology like barley, rye and triticale are known for triggering the autoimmune reaction in individuals with celiac disease. Celiac disease is an autoimmune disorder that affects the small intestine of people when they consume gluten-containing diets. The celiac disease affects about 1.0% [31] or 1.4% [32] of the world population. The storage proteins in wheat, durum, barley, rye and triticale grains, in particular prolamins, are responsible for inducing celiac disease in sensitive individuals. The celiac reaction occurs in the small intestine and damages the villi or absorptive cells resulting in malabsorption of nutrients. Currently, the only effective remedy for celiac patients is a strict lifespan adherence to a gluten-free diet. In this regard, hairless canary seed can be a potential ingredient for developing gluten-free foods especially it possesses high content of non-gluten protein (22.7 g/100 g) along with unique composition of amino acids and bioactive peptides. The absence of gluten in hairless canary seed products would not provoke the autoimmune disorder in individuals with celiac disease or sensitive to gluten.

Hairless canary seed can be considered a promising gluten-free ingredient due to its nutritional richness and functional flexibility of its flour. It can be employed for the development of food products tailored to individuals with celiac disease, gluten sensitivity or wheat allergy [4]. In fact, its utilization can help fill the nutritional gaps often seen in gluten-free foods when it comes to protein quantity and quality. It offers novel product opportunities in the growing market for allergen-friendly and health-promoting foods. Research on hairless canary seed proteins has shown the absence of gluten epitopes or amino acid sequence that provoke celiac disease [33]. In addition, the hairless canary seed proteins also exhibit sequence homology with non-gluten proteins such as rice, oat, corn and chickpea. The proteins also show no cross-reactivates with the major allergenic proteins including gluten, soy, peanuts, almonds, hazelnut, walnut, mustard, and sesame based on ELISA data. Such data demonstrate the potential of hairless canary seed flour and/or its protein fractions in developing gluten-free foods. In addition to the absence of gluten in hairless canary seed, another study has shown that the aqueous enzymatic extract from hairless canary seed at 0.5% (*w*/*w*) significantly improves gluten-free bread quality including crumb structure, loaf volume and aeration properties [34]. It is worth to mention here that hairless canary seed designated for the production of gluten-free foods should be produced and processed in a designated system that keeps the grain away from the offending grains such as wheat, to minimize the risk of cross contamination. In other words, it should be produced and processed through a dedicated system to ensure the grains are gluten-free and safe for individuals with celiac disease. Previously, a designated production system for oat production and processing was developed for the use in gluten-free foods [35].

The gluten-free foods should contain a trace level of gluten, i.e., <20 ppm (20 mg/kg) as defined by the Codex Alimentarius Commission [36]. Recent studies have shown the increasing number of celiac patients [31,32] which indicates growing demands for gluten-free foods. Additionally, the burden of celiac disease goes beyond the socio-economic impact as it also affects the healthcare cost and human productivity [37]. Undoubtedly, this indicates a need for a dietary strategy that focuses on the availability and affordability of nutritious gluten-free foods and products. In this regard, hairless canary seed flours and proteins would make a great contribution as novel food ingredients not only because of their high content of protein but also their unique quality attributes as discussed above. Hairless canary seed flours or protein fractions can be employed in a variety of gluten-free food formulations such as baked products, breakfast cereals, milk substitutes, snacks and energy bars. The absence of gluten in hairless canary seed in addition to the unique health-enhancing properties of peptides and the versatility of flour could make the grain a promising ingredient for the gluten-free food market.

## 5. Hairless Canary Seed in Diabetes and Oxidative Stress Remedies

In general, healthy eating and active lifestyle can reduce the risk of obesity and diabetes and improve the overall well-being. Obesity is a key risk factor in developing type 2 diabetes and other chronic diseases. Whole grain foods including hairless canary seed are recommended for a healthy diet due to their content of fiber, vitamins, minerals and bioactive components. In fact, several epidemiological, observational and interventional studies have shown the health benefits and protective functions of whole grain foods against obesity, diabetes, cardiovascular disease, cancer and other non-communicable diseases [38,39,40,41]. The protective mechanisms of whole grain foods against chronic diseases are not fully explored. It is believed that the grain constituents work synergistically through several mechanisms including glycemic control, antioxidant effects and anti-inflammation actions to deliver their health benefits. Hairless canary seed contains a number of bioactive components that have demonstrated health-enhancing properties such as polyphenols, carotenoids, bioactive peptides and antioxidants which could contribute to the overall health benefits of canary seed. In Mexico, canary seed is consumed as a common folk remedy for diabetes [42]. Currently, a powdery product made from canary seed is available in the market for alleviating obesity, diabetes, oxidative damage, hypertension and cardiovascular diseases. The grains are soaked in water, drained to remove the hairs, and then dried to obtain the powder product. The powder is reconstituted to make canary seed beverage prior to consumption. Without doubt the use of hairless canary seed in making food or natural health products should result in safer products with zero silica.

Bioactive peptides play significant roles in promoting human health and disease prevention through the reduction of blood sugar, enhancement of insulin sensitivity, balancing lipid metabolism, and combating inflammation and oxidative stress [43]. Studies have shown that cereal proteins are good sources of bioactive peptides that can exert beneficial health effects and combat oxidative stress in humans [44,45,46]. Peptides prepared from hairless canary seed using the standard INFOGEST protocol, have been found to exhibit equivalent or superior antioxidant and health-enhancing attributes in comparison with wheat or oat peptides based on their antioxidant, chelating, antihypertensive and antidiabetic properties [7]. Additionally, they possess potential anamnestic, antithrombotic, immuno-stimulating, opioid and neuroprotective activities. In another study, peptides derived from canary seed protein fractions including albumin, globulin, prolamin and glutelin have exhibited inhibitory effects against angiotensin-converting enzyme [47]. The IC_50_ values are 505, 443, 217 and 349 µg peptide/mL, respectively with the prolamin peptides being the most powerful antihypertensive compounds. Similar results have also been found for peptides obtained from gastrointestinal-digested canary seed milk substitute proteins [48]. The peptides derived from either canary seed flour or milk substitute (prepared by soaking the grains in water for 12 or 24 h at ambient temperature) exhibit scavenging activities against ABTS and DPPH free radicals. All the protein fractions of canary seed milk substitute peptides exhibit more antioxidant potential than that of the flours. The study identified 5 prolamin peptides derived from the 12 h soaking milk substitute having a molecular weight range of 664–1019 Da. The peptides exhibit about 74% and 44% inhibition against angiotensin-converting enzyme and dipeptidyl peptidase IV, respectively. This indicates their potential as antihypertensive and antidiabetic agents. Canary seed peptides obtained by proteolysis with Alcalase^®^ (<3 kDa and 3–10 kDa) exhibit inhibition activity against angiotensin converting enzyme (IC_50_ 0.028–0.032 mg/mL), lipase (IC_50_ 2.15–2.27 mg/mL), α-glucosidase (IC_50_ 0.82–1.15 mg/mL) and dipeptidyl-peptidase-IV (IC_50_ 1.27–1.60 mg/mL) [49]. In addition, the peptides possess high antioxidant activity against DPPH (134–151 μmol TE/mg) and ABTS (521–813 μmol TE/mg). The peptides act as mixed-type inhibitors for dipeptidyl-peptidase-IV and α-glucosidase demonstrating anti-obesity and antidiabetic activity potential [50]. Molecular docking and in silico analyses indicate that the peptides interact with angiotensin-converting enzyme as non-competitive inhibitors via destabilization of the transition state and Zn(II) coordination, while they interact as uncompetitive inhibitors against pancreatic lipase destabilizing the open-lid conformation of lipase [50]. In another study, the peptides passed through the Caco-2 monolayer indicating potential intestinal absorption [51]. The cellular antioxidant activity of canary seed peptide (<3 kDa) using Caco-2 cells is concentration-dependent with the highest activity obtained at 2.5 mg/mL. Additionally, the peptide significantly mitigates the acute and chronic oxidative damage, extending the lifespan of the nematodes by 88 and 61%, respectively using Caenorhabditis elegans model. Quantitative real-time PCR has revealed that the peptide increases the expression of oxidative stress response related gene glutathione S-transferase (GST-4) in modulating oxidative stress. In an in vivo study, mice fed normal or Western diet along with an oral dose of canary seed peptide (250 or 500 mg/kg/day), orlistat (40 mg/kg/day), or distilled water has shown that consuming canary seed peptides provide metabolic benefits, i.e., preventing weight gain by up to 20%, increasing glucose tolerance, and reducing insulin, leptin, and LDL/VLDL levels in plasma [52]. Interestingly, the dose 250 is more effective in preventing weight gain and promoting satiety than dose 500. Unexpectedly, the total ghrelin hunger hormone, which is responsible for increased appetite and more food consumption, is not affected by dose 500, but it decreases by dose 250, and increases by orlistat. The study has also shown that when canary seed extract is combined with a diet change, the rat serum triacylglycerols decreases with no significant changes in the cholesterol level [53].

Canary seed solvent extracts (chloroform, methanol and hexane) have the ability to deactivate enzymes related to obesity and type 2 diabetes [54]. The hexane extract exhibits considerable inhibition effects against several enzymes including α-amylase, α-glucosidase, rat intestinal sucrase, pancreatic lipase, lipoprotein lipase and lipolysis of 3T3-L1 adipocytes. The IC_50_ values are 2.13 and 1.25 mg/mL for α-amylase and α-glucosidase, respectively, in comparison with 0.49 and 0.68 mg/mL for Acarbose, a commonly used medication for managing type 2 diabetes. The other two solvent extracts did not show inhibitory activities against α-amylase and α-glucosidase enzymes but they inhibit lipases. The study has also demonstrated that streptozotocin nicotinamide-induced diabetic mice fed commercial canary seed milk substitute (400 mg/kg) results in reductions in serum glucose after 4 h with no side effects such as flatulence and diarrhea. The study suggests that the protection mechanism of canary seed extract is similar to that of Acarbose or Miglitol, which is based on the inhibition of α-glucosidase. In this regard, canary seed powder or extract could replace Acarbose or Miglitol as a natural health product under similar health conditions if it is proven to be safe for human use [55]. Further research on human subjects is critical to validate compatibility of canary seed extracts versus Acarbose or Miglitol. It is well known that demands for natural health products for the prevention of obesity and diabetes are of great interest due to the adverse side effects of drugs. Hairless canary seed could be a great alternative once the inhibitory effective dose is established. In a study on glycemic index of canary seed products, the flour is incorporated into wheat flour at replacement levels of 10, 30 and 50% to develop low glycemic index pancakes [56]. Using human subjects the three pancakes exhibit a low glycemic index of 34–39% that significantly decreased with the increase in canary seed flour level. Further investigation encompassing both in vitro and in vivo studies, is essential to better understand the mode of action of hairless canary seed products in the control of diabetes and obesity.

Ferulic acid (4-hydroxy-3-methoxycinnamic acid), the dominant polyphenolic compound in cereal grains including canary seed, has demonstrated several positive functions in chronic diseases including obesity and diabetes. For instance, it reduces blood glucose in streptozotocin-treated diabetic rats [57] and type 2 diabetic mice [58]. It increases plasma insulin [58] and inhibits rat maltase, sucrase and α-glucosidase [59]. Hairless canary seed contains comparable amounts of total phenolic acids to other cereal grains at level of 446 mg/kg of which ferulic acid constitutes about 63.1% followed by sinapic (21.0%) and coumaric (14.1%) [60]. In a recent study, hairless canary seed has total phenolic acid content of 325 mg/kg with ferulic acid accounting for 60.0% followed by coumaric at 33.8% [15]. In the latter study, baking hairless canary seed flour into bread and muffin results in a reduction in the bound phenolic acids and an increase in the unbound phenolic acids. Brown-seeded hairless canary seed contains relatively higher amounts of caffeic, *p*-coumaric and ferulic acids at average concentration of 102, 37 and 212 mg/kg, respectively, compared to 73, 32 and 154 mg/kg in yellow-seeded ones [61]. Both brown and yellow canary seed varieties exhibit the same flavonoid composition with flavone compounds being the main flavonoids. Germination of hairless canary seed has resulted in an increase in free, bound and total phenolic acids by about 1042, 120 and 741%, respectively compared to the ungerminated grains [62]. The study also reported higher antioxidant activities, based on DPPH, ABTS and ORAC assays, for the germinated grains in comparison with the raw grains. The level of ferulic acid (e.g., >200 mg/kg) in hairless canary seed could provoke physiological effects in humans but research is needed to demonstrate its efficacy. A recent study has shown that ferulic acid in breads and muffins (Figure 4) made from hairless canary seed alone or in blends with wheat and corn exhibits high bioaccessibility based on a simulated gastrointestinal digestion protocol but it shows very low cellular uptake by Caco-2 cell monolayer [63]. The results suggest that a small portion of ferulic acid could be absorbed in the small intestine. In addition, the processing method and conditions, and food formulations should be carefully chosen to improve bioavailability of ferulic acid by liberating large portions of bound phenolic compounds. As human studies are the benchmark and gold standard for the validation of health beneficial effects of foods, more human research on hairless canary seed foods is necessary to demonstrate the role of bioactive peptides, amino acids and phenolic acids in human health.

Human in vivo studies on hairless canary seed are currently limited. A case report published in 2023 documented the use of canary seed milk substitute in two patients with disseminated granuloma annulare, a chronic inflammatory skin condition [64]. The two patients consume two glasses of canary seed milk (2 teaspoons in 8 ounces of water) daily for 2–3 months resulting in a full recovery of their skin lesions without adverse effects [64]. The observed improvements might be due to the anti-inflammatory, antihypertensive, and antidiabetic properties of canary seed peptides such as the inhibition of DPP-IV and ACE enzymes. This case report provides preliminary insights into the potential health benefits of canary seed in humans, but it is necessary to conduct comprehensive clinical trials to substantiate the beneficial health effects and to establish safety guidelines. Another clinical trial shows that the replacement of wheat flour with canary seed flour (10–50%) in pancake formulations significantly reduces glycemic index compared with wheat pancake [56]. In general, hairless canary seed possesses several components including bioactive peptides, tryptophan, ferulic acid and lutein that could offer several protective mechanisms against chronic diseases via their roles as antioxidants, anti-inflammatory, antihypertensive, and antidiabetic agents.

## 6. Hairless Canary Seed and Health of Eyes and Brain

Lutein plays significant roles in promoting the health of eyes [65,66] and brain [67,68] and in reducing the risk of age-related macular degeneration [69], cataracts [70], breast cancer [71], and cardiovascular disease [72]. These beneficial health effects could be provoked via multiple physiological functions of lutein in humans, e.g., scavenging reactive oxygen species, binding physiological proteins, and absorbing harmful blue light among others. Hairless canary seed, corn, durum and einkorn wheat grains are considered good sources of carotenoids compared to other cereal grains with lutein being the predominant carotenoid [14,73]. These grains can be incorporated into common foods to boost the daily consumption of lutein, especially the daily intake of lutein is extremely lower (1.0–1.8 mg/day) [74] than that of the suggested effective dose of 6 mg/day based on reducing the risk of eye diseases [75]. Currently, no dietary reference intake for lutein is available in spite of its role in the health of eye and brain during the lifespan of humans.

A recent study identified the thorough composition of carotenoids in hairless canary seed and their changes during bread- and muffin-making processes [14]. The study identified 19 carotenoid compounds in hairless canary seed with all-*trans* lutein being the predominant carotenoid, and lutein 3-*O*-linoleate comes second followed by lutein 3-*O*-oleate and lutein di-linoleate. The study reported that hairless canary seed is fairly rich in carotenoids with a total content of 7.6 µg/g in comparison with 1.3 µg/g in common wheat and 12.9 µg/g in corn. Table 1 shows the main carotenoids in muffins and breads baked from hairless canary seed alone (Figure 4) or from blends of hairless canary seed and corn. The study reported significant reductions in carotenoids in muffin and bread products after baking (Table 1). The study suggests that batter or dough preparation causes more reductions in carotenoids than oven baking due to enzymatic oxidation and degradation. Furthermore, muffin-making process causes lower lutein reductions compared with bread-making process. It appears that muffins made from hairless canary seed alone or in blends with corn could boost the daily intake of lutein and zeaxanthin. The bioaccessibility of lutein from muffins is relatively high being 81–94% but its cellular uptake is low (7–9%) based on the use of simulated gastrointestinal digestion protocol combined with Caco-2 cell model [63]. The bioaccessibility of zeaxanthin (lutein isomer) is lower than lutein probably due to its lower concentration in muffins but having close cellular uptake of 4–11%. Breads made from wheat and hairless canary seed blends (Figure 4) exhibit higher lutein bioaccessibility (47–80%) than the control bread (42%) with apical uptake of 4.3–9.2%. Similar to muffin, the bioaccessibility of zeaxanthin from breads is lower than lutein. In general, hairless canary seed muffins or hairless canary seed/corn muffins are good sources of lutein but a small portion could be absorbed in the small intestine. Cookies, muffins, and flatbreads made from ancient einkorn wheat enriched with lutein have been found to hold the potential to improve lutein intake based on in vitro study that simulates the human upper gastrointestinal digestion coupled with Caco-2 cells monolayer absorption [76]. The study also indicates that the fed state conditions exhibit significantly higher lutein bioaccessibility compared with the fasted state conditions. The bioaccessibility of cookies and muffins is higher than that of flatbreads due to their higher content of fat content, measured under either fed or fasted conditions. These preliminary studies have shown that foods made from hairless canary seed, einkorn and corn alone or in blends could boost the daily consumption of lutein, lutein esters and other carotenoids present in the grains.

## 7. Conclusions

The entire removal of the harmful silicious spicules makes hairless canary seed a safe ingredient for making food and natural health products. It is free from silica and safe for human consumption based on the thorough assessment by US-FDA and Health Canada. Hairless canary seed is a true cereal grain that holds not only a unique nutritional profile but also exceptional functional properties. The unique nutrient composition of hairless canary seed such as its high content of non-gluten protein, quality of starch and unsaturated oil along with special functional characteristics would make it a potential functional ingredient in the development of natural heath products and functional foods. These characteristics also makes the grain an excellent choice for dietary strategies aiming at supporting heathy eating and health promotion particularly in the areas of oxidative stress, diabetes, vision and cognition. Hairless canary seed possesses several components including bioactive peptides, tryptophan, ferulic acid and lutein that could offer several protective mechanisms against chronic diseases via their roles as antioxidants, anti-inflammatory, antihypertensive, and antidiabetic agents. The grain also holds potential for the development of gluten-free foods and products designed for celiac patients. More research in the area of product development for specific end uses is needed in order to help expand the hairless canary seed food market. Additionally, more in vitro and in vivo studies are essential to pave the way for hairless canary seed into the functional food and natural health product industries.

## Figures and Tables

**Table 1:** Main carotenoids in muffin and bread products (µg/g) and the impact of baking process on composition of carotenoids (% decrease).

Carotenoids	Muffin			Bread		
	HCS (100%)	HCS/C (1:1, *w*/*w*)	HCS/C (1:2, *w*/*w*)	Wheat (100%)	Wheat/HCS (75/25, *w*/*w*)	Wheat/HCS (50/50, *w*/*w*)
15-*cis*-Lutein	0.05	0.07	0.1	0.04	0.03	0.06
all-*trans*-Lutein	1.5	1.7	2.0	0.3	0.7	0.8
all-*trans*-Zeaxanthin	0.2	1.5	2.2	0.2	0.2	0.3
9-*cis*-Lutein	0.2	0.1	0.1	nd	nd	nd
9-*cis*-Zeaxanthin	0.1	0.1	0.2	nd	nd	nd
15-*cis-*β-Cryptoxanthin	0.2	0.2	0.2	nd	nd	nd
all-*trans*-β-Cryptoxanthin	0.1	0.2	0.2	nd	nd	nd
Lutein-3-*O*-linoleate	1.0	0.4	0.5	nd	0.4	0.6
Lutein-3-*O*-oleate	0.3	0.4	0.2	nd	0.3	0.4
Lutein dilinoleate	0.2	0.1	0.1	nd	0.3	0.4
Total unbounds (free)	2.4	3.9	5.0	0.5	0.9	1.2
Total bounds (mono- and di-esters)	1.5	0.9	0.8	0.0	1.0	1.4
Total carotenoids	3.9	4.8	5.8	0.5	1.9	2.6
Impact of baking process expressed as % decrease						
Total unbounds	44.2 (18.6) ^a^	55.7 (13.7)	50.0 (13.0)	66.7 (20.0)	59.1 (4.6)	52.0 (4.0)
Total bounds	53.1 (15.6)	40.0 (13.3)	38.5 (7.7)	-	37.5 (12.5)	41.7 (12.5)
Total carotenoids	48.0 (17.3)	53.4 (13.6)	48.7 (12.4)	66.7 (20.0)	50.0 (7.9)	46.9 (8.1)

^a^ Figures between brackets are the reduction percent due to oven baking. nd: Not Detected. Source: Abdel-Aal et al., 2022 [14].

## Data Availability

No new data were created or analyzed in this study. Data sharing is not applicable to this article.

## References

[1] Food and Agriculture Organization of the United Nation (FAO) FAO Stat: Crops and Livestock Products, 2023. Update 20 December 2024. https://www.fao.org/faostat/en/#data/QCL.

[2] Abdel-Aal E.M., Hucl P., Sosulski F.W. (1997). Structural and compositional characteristics of canaryseed (*Phalaris canariensis* L.). J. Agric. Food Chem..

[3] O’Neill C.H., Hodges G.M., Riddle P.N., Jordan P.W., Newman R.H., Flood R.J., Toulson E.C. (1980). A fine fibrous silica contaminant of flour in the high esophageal cancer area of north-east Iran. Int. J. Cancer.

[4] Abdel-Aal E.M. (2021). Nutritional and functional attributes of hairless canary seed groats and components and their potential as functional ingredients. Trends Food Sci. Technol..

[5] US-FDA 2015. US Food and Drug Administration. Agency Response Letter GRAS Notice No. GRN 000529. https://www.hfpappexternal.fda.gov/scripts/fdcc/index.cfm?set=grasnotices&id=529.

[6] Health Canada 2016 Health Canada, Food and Nutrition, Novel Foods. http://www.hc-sc.gc.ca/fn-an/gmf-agm/appro/canary-seed-lang-graine-alpiste-eng.php.

[7] Mason E., L’Hocine L., Achouri A., Pitre M., Karboune S. (2020). Health promoting bioactive properties of novel hairless canary seed flour after in vitro gastrointestinal digestion. Foods.

[8] Abdel-Aal E.M., Hernandez M., Rabalski I., Hucl P. (2020). Composition of hairless canary seed oil and starch-associated lipid and its relationship to pasting and thermal properties of starch. LWT—Food Sci. Technol..

[9] Ben Salah H., Kchaou M., Ben Abdallah Kolsi R., Abdennabi R., Ayedi M., Gharsallah N., Allouche N. (2018). Chemical composition, characteristics, profiles and bioactivities of Tunisian *Phalaris canariensis* seed: A potential source of ω-6 and ω-9 fatty acids. J. Oleo Sci..

[10] Abdel-Aal E.M., Rabalski I., Hernandez M., L’Hocine L., Patterson C.A., Hucl P. (2019). Effect of sodium chloride, sucrose, and xanthan gum on pasting properties and gel syneresis of hairless canary seed starch. Cereal Chem..

[11] Irani M., Abdel-Aal E.M., Razavi S.M.A., Hucl P., Patterson C.A. (2017). Thermal and functional properties of hairless canary seed (*Phalaris canariensis* L.) starch in comparison with wheat starch. Cereal Chem..

[12] Irani M., Razavi S.M.A., Abdel-Aal E.M., Hucl P., Patterson C.A. (2019). Viscoelastic and textural properties of canary seed starch gels in comparison with wheat starch gel. Int. J. Biol. Macromol..

[13] Abdel-Aal E.M., Hucl P., Miller S.S., Patterson C.A., Gray D. (2011). Microstructure and nutrient composition of hairless canary seed and its potential as a blending flour for food use. Food Chem..

[14] Abdel-Aal E.M., Mats L., Rabalski I. (2022). Identification of carotenoids in hairless canary seed and the effect of baking on their composition in bread and muffin products. Molecules.

[15] Abdel-Aal E.M., Rabalski I. (2022). Changes in phenolic acids and antioxidant properties during baking of bread and muffin made from blends of hairless canary seed, Wheat, and Corn. Antioxidant.

[16] Amahrous A., Taib M., Meftah S., Oukani E., Lahboub B. (2024). Chemical composition, health benefits and future prospects of hairless canary seed (*Phalaris canariensis* L.): A Review. J. Oleo Sci..

[17] Patterson C.A., Malcolmson L., Lukie C., Young G., Hucl P., Abdel-Aal E.M. (2018). Glabrous canary seed: A novel food ingredient. Cereal Foods World.

[18] Abdel-Aal E.M., Hucl P., Abdel-Aal E.M., Wood P. (2005). Hairless canary seed: A potential food crop. Specialty Grains for Food and Feed.

[19] Abdel-Aal E.M., Hucl P., Patterson C.A., Gray D. (2010). Fractionation of hairless canary seed (*Phalaris canariensis* L.) into starch, protein and oil. J. Agric. Food Chem..

[20] Agus A., Planchais J., Sokol H. (2018). Gut microbiota regulation of tryptophan metabolism in health and disease. Cell Host Microbe.

[21] Yao K., Fang J., Yin Y.-l., Feng Z.-M., Tang Z.-R., Wu G. (2011). Tryptophan metabolism in animals: Important roles in nutrition and health. Front. Biosci..

[22] Nayak B.N., Singh R.B., Buttar H.S. (2019). Role of tryptophan in health and disease: Systematic review of the anti-oxidant, anti-inflammation, and nutritional aspects of tryptophan and its metabolites. World Heart J..

[23] Passos C.S., Carvalho L.N., Pontes R.B., Campos R.R., Ikuta O., Boim M.A. (2012). Blood pressure reducing effects of *Phalaris canariensis* in normotensive and spontaneously hypertensive rats. Can. J. Physiol. Pharmacol..

[24] Venkatesh R., Srinivasan K., Singh S.A. (2017). Effect of arginine:lysine and glycine:methionine intake ratios on dyslipidemia and selected biomarkers implicated in cardiovascular disease: A study with hypercholesterolemic rats. Biomed. Pharmacother..

[25] Newkirk R.W., Ram J.I., Hucl P., Patterson C.A., Classen H.L. (2011). A study of nutrient digestibility and growth performance of broiler chicks fed hairy and hairless canary seed (*Phalaris canariensis* L.) products. Poult. Sci..

[26] Classen H., Cho M., Hucl P., Gomis S., Patterson C.A. (2014). Performance, health and tissue weights of broiler chickens fed graded levels of hairless hulled yellow and brown canary seed (*Phalaris canariensis* L.). Can. J. Anim. Sci..

[27] Thacker P.A. (2003). Performance and carcass characteristics of growing-finishing pigs fed diets containing graded levels of canaryseed. Can. J. Anim. Sci..

[28] L’Hocine L., Achouri A., Mason E., Pitre M., Martineau-Côté D., Sirois S., Karboune S. (2023). Assessment of protein nutritional quality of novel hairless canary seed in comparison to wheat and oat using in vitro static digestion models. Nutrients.

[29] Rajamohamed S.H., Aryee A.N.A., Hucl P., Patterson C.A., Boye J.I. (2013). In vitro gastrointestinal digestion of glabrous canaryseed proteins as affected by variety and thermal treatment. Plant Foods Hum. Nutr..

[30] Moura M.A.F., Perera S., Ren Y., Takahashi J.A., Ai Y., Nickerson M.T. (2020). Functional characteristics and protein quality of selected commercially obtained brown and yellow canary seed flours and prepared isolates. Cereal Chem..

[31] Kurppa K., Mulder C.J., Stordal K., Kaukinen K. (2024). Celiac disease affects 1% of global population: Who will manage all these patients?. Gastroenterology.

[32] Singh P., Arora A., Strand T.A., Leffler D.A., Catassi C., Green P.H., Kelly C.P., Ahuja V., Makharia G.K. (2018). Global prevalence of celiac disease: Systematic review and meta-analysis. Clin. Gastroenterol. Hepatol..

[33] Boye J.I., Achouri A., Raymond N., Cleroux C., Weber D., Koerner T.B., Hucl P., Patterson C.A. (2013). Analysis of glabrous canary seeds by ELISA, mass spectrometry, and western blotting for the absence of cross-reactivity with major plant food allergens. J. Agric. Food Chem..

[34] Dios Sanz E., Sanmartino T., Campderrós M.E., Rodriguez Furlán L.T. (2024). Obtaining and evaluating of enzymatic extracts from hairless canary seed (CDC Maria) as gluten-free bread-improving agents. J. Food Sci. Technol..

[35] Burrows V.D., Abdel-Aal E.M., Wood P. (2005). Hulless oats. Specialty Grains for Food and Feed.

[36] (2008). Draft Revised Codex Standard for Foods for Special Dietary Use for Persons Intolerant to Gluten.

[37] Herrera-Quintana L., Navajas-Porras B., Vázquez-Lorente H., Hinojosa-Nogueira D., Corrales-Borrego F.J., Lopez-Garzon M., Plaza-Diaz J. (2025). Celiac disease: Beyond diet and food awareness. Foods.

[38] Bjorck I., Ostman E., Kristensen M., Anson N.M., Price R.K., Haenen G.R.M.M., Havenaar R., Knudson K.E.B., Frid A., Mykkanen H. (2012). Cereal grain for nutrition and health benefits: An overview of results in vitro, animal and human studies in health grain project. Trends Food Sci. Technol..

[39] Jones J.M., García C.G., Braun H.J. (2020). Perspective: Whole and Refined Grains and Health-Evidence Supporting “Make Half Your Grains Whole”. Adv. Nutr..

[40] Sang S., Idehen E., Zhao Y., Chu Y.F. (2020). Emerging science on whole grain intake and inflammation. Nutr. Rev..

[41] Seal C.J., Brownlee I.A. (2010). Whole grains and health, evidence from observational and intervention studies. Cereal Chem..

[42] Perez Gutierrez R.M., Ahuatzi D.M., Horcacitas M.C., Baez E.F., Victoria T.C., Motaflores J.M. (2014). Ameliorative effect of hexane extract of *Phalaris canariensis* on high fat diet-induced obese and streptozotocin-induced diabetic mice. Evid. Based Complement. Altern. Med..

[43] Yu J., Chen G., Jin Y., Zhang M., Wu T. (2025). Research progress of bioactive peptides in improving type II diabetes. Foods.

[44] Esfandi R., Walters M.E., Tsopmo A. (2019). Antioxidant properties and potential mechanisms of hydrolyzed proteins and peptides from cereals. Heliyon.

[45] Gong X., An Q., Le L., Geng F., Jiang L., Yan J., Wan Y. (2020). Prospects of cereal protein-derived bioactive peptides: Sources, bioactivities diversity, and production. Crit. Rev. Food Sci. Nutr..

[46] Wu Q., Guo Z., Zhou Z., Jin M., Li Q., Zhou X. (2022). Recent advances in bioactive peptides from cereal-derived foodstuffs. Int. J. Food Sci. Nutr..

[47] Valverde M.E., Orona-Tamayo D., Nieto-Rendon B., Paredes-Lopez O. (2017). Antioxidant and antihypertensive potential of protein fractions from flour and milk substitutes from canary seeds (*Phalaris canariensis* L.). Plant Food Hum. Nutr..

[48] Estrada-Salas P.A., Montero-Moran G.M., Martinez-Cuevas P.P., Gonzalez C., Barba de la Rosa A.P. (2014). Characterization of antidiabetic and antihypertensive properties of canary seed (*Phalaris canariensis* L.) peptides. J. Agric. Food Chem..

[49] Urbizo-Reyes U.C., Aguilar-Toalá J.E., Liceaga A.M. (2021). Hairless canary seeds (*Phalaris canariensis* L.) as a potential source of antioxidant, antihypertensive, antidiabetic, and antiobesity biopeptides. Food Prod. Process. Nutr..

[50] Urbizo-Reyes U., Liceaga A.M., Reddivari L., Li S., Kim K.H., Anderson J.M. (2022). Enzyme kinetics, molecular docking, and in silico characterization of canary seed (*Phalaris canariensis* L.) peptides with ACE and pancreatic lipase inhibitory activity. J. Funct. Foods.

[51] Urbizo-Reyes U., Kim K.-H., Reddivari L., Anderson J.M., Liceaga A.M. (2022). Oxidative stress protection by canary seed (*Phalaris canariensis* L.) peptides in Caco-2 cells and Caenorhabditis elegans. Nutrients.

[52] Urbizo-Reyes U., Liceaga A.M., Reddivari L., Li S., Kim K.H., Cox A.D., Anderson J.M. (2022). Canary seed (*Phalaris canariensis* L.) peptides prevent obesity and glucose intolerance in mice fed a Western diet. Int. J. Mol. Sci..

[53] Ojeda L., Ruiz Y.D., Martínez F., Odreman R., Torri J., Villegas R., Ybarra L.P., Machado N.N. (2021). Effects of the seed of *Phalaris Canariensis* and the changes of diet on serum lipids in rats. Emir. J. Food Agric..

[54] Perez Gutierrez R.M., Ahuatzi D.M., Victoria T.C. (2016). Inhibition by seeds of *Phalaris canariensis* extracts of key enzymes linked to obesity. Altern. Ther. Health Med..

[55] Rayburn K. (2016). Canary seed in diabetes: Sweet harmony?. Altern. Ther. Health Med..

[56] Escalante-Figueroa F., Castellanos-Ruelas A., Castañeda-Pérez E., Chel-Guerrero L., Betancur-Ancona D. (2024). Development of low glycemic index pancakes formulated with canary seed (*Phalaris canariensis*) Flour. Plant Foods Hum. Nutr..

[57] Prabhakar P.K., Prasad R., Ali S., Doble M. (2013). Synergistic interaction of ferulic acid with commercial hypoglycemic drugs in streptozotocin induced diabetic rats. Phytomedicine.

[58] Jung E.H., Kim S.R., Hwang I.K., Ha T.Y. (2007). Hypoglycemic effects of a phenolic acid fraction of rice bran and ferulic acid in C57BL/Ks]-db/db mice. J. Agric. Food Chem..

[59] Adisakwattana S., Chantarasinlapin P., Thammarat H., Yibchok-Anun S. (2009). A series of cinnamic acid derivatives and their inhibitory activity on intestinal alpha-glucosidase. J. Enzyme Inhib. Med. Chem..

[60] Abdel-Aal E.M., Hucl P., Patterson C.A., Gray D. (2011). Phytochemicals and heavy metals content of hairless canary seed: A variety developed for food use. LWT—Food Sci. Technol..

[61] Li W., Qiu Y., Patterson C.A., Beta T. (2011). The analysis of phenolic constituents in glabrous canaryseed groats. Food Chem..

[62] Chen Z., Yu L., Wang X., Gu Z., Beta T. (2016). Changes of phenolic profiles and antioxidant activity in canaryseed (*Phalaris canariensis* L.) during germination. Food Chem..

[63] Abdel-Aal E.M., Rabalski I., Carey C., Gamel T.H. (2023). Bioaccessibility and cellular uptake of lutein, zeaxanthin and ferulic acid from muffins and breads made from hairless canary seed, wheat and corn blends. Foods.

[64] Park L., Green C., Arutyunyan S., Vasile G., Buckley C., Weiss E. (2023). Effects of canary seed on two patients with disseminated granuloma annulare. Dermatol. Rep..

[65] Abdel-Aal E.M., Akhtar M.H., Zaheer K., Rashida A. (2013). Dietary sources of lutein and zeaxanthin carotenoids and their role in eye health. Nutrients.

[66] Johnson E.J. (2014). Role of lutein and zeaxanthin in visual and cognitive function throughout lifespan. Nutr. Rev..

[67] Li J., Abdel-Aal E.M. (2021). Dietary lutein and cognitive function in adults: A Meta-analysis of randomized controlled trials. Molecules.

[68] Stringham J.M., Johnson E.J., Hammond B.R. (2019). Lutein across the lifespan: From childhood cognitive performance to the aging eye and brain. Curr. Dev. Nutr..

[69] Chew E.Y., Clemons T.E., Agrón E., Launer L.J., Grodstein F., Bernstein P.S. (2015). Effect of omega-3 fatty acids, lutein/zeaxanthin, or other nutrient supplementation on cognitive function: The AREDS2 randomized clinical trial. JAMA.

[70] Jia Y.P., Sun L., Yu H.S., Liang L.P., Li W., Ding H., Song X.B., Zhang L.J. (2017). The pharmacological effects of lutein and zeaxanthin on visual disorders and cognition diseases. Molecules.

[71] Gong X., Smith J.R., Swanson H.M., Rubin L.P. (2018). Carotenoid lutein selectively inhibits breast cancer cell growth and potentiates the effect of chemotherapeutic gents through ROS-mediated mechanisms. Molecules.

[72] Leermakers E.T.M., Darweesh S.K.L., Baena C.P., Moreira E.M., Melo van Lent D., Tielemans M.J., Muka T., Vitezova A., Chowdhury R., Bramer W.M. (2016). The effects of lutein on cardiometabolic health across the life course: A systematic review and meta-analysis. AJCN.

[73] Abdel-Aal E.M., Young J.C., Rabalski I., Frégeau-Reid J., Hucl P. (2007). Identification and quantification of seed carotenoids in selected wheat species. J. Agric. Food Chem..

[74] Johnson E.J. (2010). Intake of lutein and zeaxanthin differ with age, sex, and ethnicity. J. Am. Diet. Assoc..

[75] Seddon J.M., Ajani U.A., Sperduto R.D., Hiller R., Blair N., Burton T.C., Farber M.D., Gragoudas E.S., Haller J., Miller D.T. (1994). Dietary carotenoids, vitamin A, C and E, and advanced age-related macular degeneration. JAMA.

[76] Read A., Wright A., Abdel-Aal E.M. (2015). In vitro bioaccessibility and monolayer uptake of lutein from whole grain baked foods. Food Chem..

